# Oral Transmission of *Trypanosoma cruzi,* Brazilian Amazon

**DOI:** 10.3201/eid2501.180646

**Published:** 2019-01

**Authors:** Rosa Amélia G. Santana, Maria Graças V.B. Guerra, Débora R. Sousa, Kátia Couceiro, Jessica V. Ortiz, Maurício Oliveira, Lucas S. Ferreira, Kenny R. Souza, Igor C. Tavares, Romulo F. Morais, George A.V. Silva, Gisely C. Melo, Gabriel M. Vergel, Bernardino C. Albuquerque, Ana Ruth L. Arcanjo, Wuelton M. Monteiro, João Marcos B.B. Ferreira, Marcus V.G. Lacerda, Henrique Silveira, Jorge Augusto O. Guerra

**Affiliations:** Fundação de Medicina Tropical Dr Heitor Vieira Dourado, Manaus, Brazil (R.A.G. Santana, M.G.V.B. Guerra, D.R. Sousa, K. Couceiro, J.V. Ortiz, M. Oliveira, L.S. Ferreira, G.A.V. Silva, G.C. Melo, G.M. Vergel, W.M. Monteiro, M.V.G. Lacerda, J.A.O. Guerra);; Universidade do Estado do Amazonas, Manaus (R.A.G. Santana, M.G.V.B. Guerra, D.R. Sousa, K. Couceiro, J.V. Ortiz, M. Oliveira, K.R. Souza, I.C. Tavares, R.F. Morais, G.C. Melo, W.M. Monteiro, J.M.B.B. Ferreira, J.A.O. Guerra);; Fundação de Vigilância em Saúde do Amazonas, Manaus (G.M. Vergel, B.C. Albuquerque, A.R.L. Arcanjo);; Instituto Leônidas & Maria Deane, Fiocruz, Manaus (M.V.G. Lacerda);; Instituto de Higiene e Medicina Tropical, Universidade Nova de Lisboa, Lisbon, Portugal (H. Silveira)

**Keywords:** Chagas disease, *Trypanosoma cruzi*, parasites, *Euterpe oleracea*, açaí juice, *Triatominae*, food safety, disease outbreak, oral transmission, Brazilian Amazon, Brazil

## Abstract

In the Brazilian Amazon, the suspected source of infection in an outbreak of acute Chagas disease involving 10 patients was *Euterpe oleracea* (açaí berry) juice. Patient blood and juice samples contained *Trypanosoma cruzi* TcIV, indicating oral transmission of the Chagas disease agent.

In Latin America, Chagas disease is prevalent in 21 countries and is one of the most worrisome public health problems on the subcontinent. The social and economic effects among poor and neglected populations are high (*1*). Increasing reports of outbreaks of acute Chagas disease have come to the attention of public health authorities, who regard the disease as emerging in the Amazon (*2*). For most reported outbreaks, epidemiologic investigation points to nonvectored transmission, implicating juices from local fruits (*3*). A major suspected source of infection is *Euterpe oleracea*, the açaí berry, consumed widely as a drink made from a blended pulp (*4*).

## The Study

On December 29, 2017, a 51-year-old woman with acute febrile syndrome visited a tertiary care center for infectious diseases (Fundação de Medicina Tropical Dr Heitor Vieira Dourado; FMT-HVD) in Manaus, the capital of Amazonas state, Brazil, where malaria is endemic. A routine thick blood smear was negative for *Plasmodium* spp. but positive for *Trypanosoma cruzi* trypomastigotes. The patient mentioned 3 sick relatives in Manaus and 6 more in Lábrea, a municipality 850 km south of Manaus, where she visited often (Table). A common exposure factor among them was ingestion of açaí berry juice, produced by local dealers in the outskirts of Lábrea and sent to Manaus for consumption. Thick blood smears from the other 9 patients, all with acute febrile syndrome, were positive for *T. cruzi*. Of these 10 patients, 8 were clinically assessed at FMT-HVD and submitted samples for direct xenodiagnosis and peripheral blood for *T. cruzi* culture and PCR. A sample of the same juice drunk by all the patients, maintained at −20°C in the family refrigerator in Lábrea, was collected by local health authorities and sent to the reference laboratory in Manaus. All patients with a diagnosis of acute Chagas disease were prescribed benznidazole for 60 days (*5*).

**Table:** Basic demographics and diagnostic methods used to confirm acute Chagas disease in 10 patients, Brazilian Amazon*

Patient	Patient age, y/sex	Date of diagnosis	Blood smear	Xenodiagnosis	Culture
1	51/F	2017 Dec 29	+	+	+
2	19/F	2017 Dec 29	+	+	+
3	22/M	2017 Dec 29	+	+	+
4	35/F	2018 Jan 5	+	+	+
5	1/F	2018 Jan 5	+	NP	+
6	21/F	2018 Dec 1	+	+	–
7	65/F	2018 Dec 1	+	+	+
8	16/F	2018 Dec 1	–	+	NP
9	11/M	2018 Dec 1	+	NP	NP
10	51/F	2018 Dec 1	+	NP	NP

*NP, not performed; +, positive for *Trypanosoma cruzi*; –, negative for *T. cruzi*.

Blood samples were obtained by venipuncture from 8 of the 10 patients, and ≈10 mL of blood was collected into heparin-containing tubes. Next, 100 μL whole blood was distributed into 3 mL liver infusion tryptose medium containing 20% inactivated fetal calf serum and 40 mg/mL gentamycin sulfate and then incubated at 27°C. Inverted optical microscopy was used daily to search for flagellate forms. Xenodiagnosis was conducted by using 20 stage 3 nymphs of *Rhodnius robustus* and *R. prolixus* bugs. We centrifuged 5 μL blood at 4,000 rpm for 10 min and collected the buffy coat for DNA extraction by using the PureLink Kit (Invitrogen, https://www.thermofisher.com).

**Figure 1:**
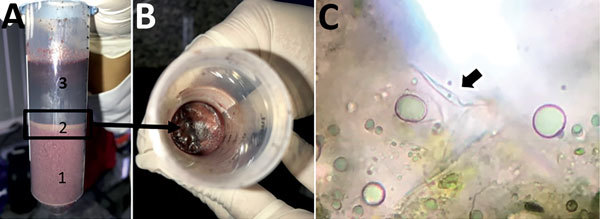
Zçaí berry juice sample from Brazilian Amazon. A) A 50 mL-tube after centrifugation shows 3 layers: 1, pulp, 2, intermediate fat (box); and 3: supernatant. B) Top view of layer 2 (arrow). C) Fresh preparation of layer 2 showing *Trypanosoma cruzi* flagellated form (arrow).

On December 11, a sample of the açaí juice was transported on ice to FMT-HVD, where it was immediately thawed and centrifuged in 50-mL tubes at 3,000 rpm for 5 minutes. After centrifugation, 3 layers were observed: pulp, an intermediate layer containing fat, and supernatant (Figure 1, panels A and B). From each phase, we suspended 300 μL into 1 mL liver infusion tryptose medium containing 20% inactivated fetal calf serum and 40 mg/mL gentamycin sulfate and incubated it at 27°C. We made triplicate cultures and used inverted optical microscopy to search daily for flagellate forms.

We placed 200 μL of the açaí culture in 1.5-mL microtubes with 500 μL of phosphate-buffered saline at pH 7.2, incubated the sample in a water bath for 15 minutes at 98°C, and centrifuged it at 3,500 rpm for 3 minutes. We removed 200 μL of supernatant for genotyping.

The nontranscribed spacer of the mini-exon gene was amplified according to the multiplex protocol described elsewhere (*6*). The 150-bp product is characteristic of *T. cruzi* zymodeme Z3 of discrete typing units (DTUs) TcIII or TcIV; 100 bp is characteristic of *T. rangeli*, 200 bp of *T. cruzi* TcI, and 250 bp of *T. cruzi* TcII. Mini-exon gene analysis cannot distinguish between TcIII and TcIV.

All samples were also subjected to mitochondrial and nuclear DNA typing by analyzing polymorphisms in the cytochrome oxidase subunit II (COII) (*7*) and the glucose-phosphate isomerase (GPI) (*8*) genes, respectively. The amplified PCR products were purified by using the Wizard SV Gel and PCR Clean-up System kit (Promega, https://www.promega.com.br) and sequenced. Sequencing was performed with an ABI 3130 DNA sequencer (Applied Biosystems, https://www.thermofisher.com). We followed the BigDye Terminator v3.1 Cycle Sequencing Kit protocol (Applied Biosystems) by using 10–40 ng of purified PCR product in the sequencing reaction and the same primers used for COII and GPI gene amplification by PCR. We used sequences from standard strains: TcI (Silvio X10 cl1), TcII (Esmeraldo cl3), TcIII (M6241 cl6), TcIV (CANIII cl1), TcV (Mn cl2), and TcVI (CL Brener). Maximum-likelihood phylogenetic trees were inferred by using W-IQ-TREE (*9*).

During the outbreak, 8 patients who had drunk the açaí juice were clinically assessed at FMT-HVD. Parasite culture was successful for 6 and xenodiagnoses for 7. A total of 5 *T. cruzi* strains were isolated by blood culture and xenodiagnosis. All 8 patients were *T. cruzi* positive by PCR. Blood culture, xenodiagnoses, and PCR were not performed for 2 patients because they did not attend follow-up at FMT-HVD; their diagnoses were based only on thick blood smears.

**Figure 2:**
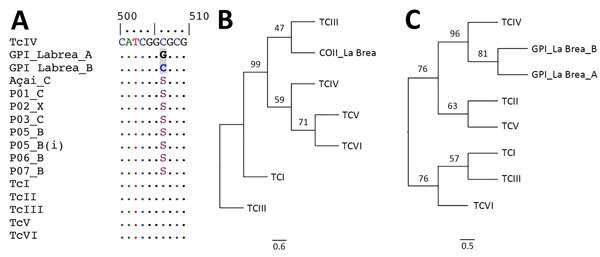
Comparison of *Trypanosoma cruzi* açaí juice samples and Chagas disease patient blood samples, Brazilian Amazon. A) Alignment of GPI sequences from açaí juice samples and patient blood samples. B–C) Phylogenetic position of *T. cruzi* responsible for the 2017 Chagas disease outbreak in the Brazilian Amazon, based on the cytochrome oxidase subunit II gene sequences (best-fit model: Hasegawa-Kishino-Yano) and on the GPI gene sequences (best-fit model: Kimura 2-parameter). The following standard strains obtained from GenBank (discrete typing units [strain name, access no. for COII–GPY]) were used: TcI (SilvioX10 cl4, EU302222.1–Silvio10cl1–AY540730.1), TcII (Esmeraldo cl3, AF359035.1–AY540728.1), TcIII (M6241 cl6, AF359032.1– AY484478.1), TcIV (CANIII cl1–AF359030.1), TcV (Mn cl2, DQ343718.1–AY484480.1), and TcVI (CL Brener, DQ343645.1–XM_815802.1). B, blood; C, culture; GPI, glucose-phosphate isomerase; P0, patient number; X, xenodiagnosis; (i) repetition number. Scale bars indicate number of mutations per site.

After 24 h of incubation, we observed flagellated motile forms in the intermediate layer of fat of centrifuged açaí juice (Figure 1, panel C). *T. cruzi* from human samples and açaí juice showed an identical 150-bp band of mini-exon compatible with *T. cruzi* zymodeme Z3 (*6*), consistent with COII and GPI sequencing results. Parasites differed in position 507 (G/C) of the GPI sequence (Figure 2, panel A). COII sequences were compatible with *T. cruzi* III mitochondrial ancestral lineage (*7*). This set of samples and the reference strains TcIII, TcIV, TcV, and TcVI formed a single cluster that shared a characteristic mitochondrial genome distinct from both TcI and TcII (Figure 2, panel B). GPI sequence analysis showed that the human blood and açaí juice *T. cruzi* samples could be consistently classified as TcIV DTU (*8*) (Figure 2, panel C). Alignments of sequences from COII and GPI *T. cruzi* genes showed that the parasites in the açaí juice were the same.

## Conclusions

All patients who simultaneously exhibited febrile syndrome had consumed açaí juice from the same source in the previous weeks. They were all infected with the same *T. cruzi* DTU as that in the juice, strongly suggestive that in the Brazilian Amazon, contaminated açaí juice is a source of oral contamination with *T. cruzi*. Circumstantial association between outbreaks and contaminated açaí juice has been suggested by previous studies from South America (*10*–*12*), but our evidence of an acute Chagas disease outbreak in which oral transmission could be implicated is robust because patients and a sample of the juice consumed were analyzed in a paired manner.

The most probable hypothesis for contamination of the juice is based on the attraction of contaminated triatomines by the light used during nighttime açaí pulp extraction. Another hypothesis is that contamination occurred during collection and manipulation of açaí berries without use of proper hygiene before mashing (*1*). Triatomine infestation of Amazonian palm trees also supports the potential for oral *T. cruzi* contamination of humans (*13*).

Experimental contamination of food with *T. cruzi* shows that parasite survival varies with the type of food and presence or absence of refrigeration (*14*,*15*). In this study, we hypothesized that the long survival of the parasite is associated with freezing the sample in the presence of cryoprotectants probably present in the fatty content of the açaí juice. On the basis of our demonstration of this route of contamination, legislation should be revised to possibly require pasteurization of açaí juice (*14*).
